# Patient engagement with infection management in secondary care: a qualitative investigation of current experiences

**DOI:** 10.1136/bmjopen-2016-011040

**Published:** 2016-10-31

**Authors:** Timothy M Rawson, Luke S P Moore, Bernard Hernandez, Enrique Castro-Sanchez, Esmita Charani, Pantelis Georgiou, Raheelah Ahmad, Alison H Holmes

**Affiliations:** 1 National Institute for Health Research Health Protection Research Unit in Healthcare Associated Infections and Antimicrobial Resistance, Imperial College London, London UK; 2 Imperial College Healthcare NHS Trust, London, UK; 3 Centre for Bio-Inspired Technology, Imperial College London, London, UK

**Keywords:** INFECTIOUS DISEASES, antimicrobials, shared decision making

## Abstract

**Objective:**

To understand patient engagement with decision-making for infection management in secondary care and the consequences associated with current practices.

**Design:**

A qualitative investigation using in-depth focus groups.

**Participants:**

Fourteen members of the public who had received antimicrobials from secondary care in the preceding 12 months in the UK were identified for recruitment. Ten agreed to participate. All participants had experience of infection management in secondary care pathways across a variety of South-East England healthcare institutes. Study findings were subsequently tested through follow-up focus groups with 20 newly recruited citizens.

**Results:**

Participants reported feelings of disempowerment during episodes of infection in secondary care. Information is communicated in a unilateral manner with individuals ‘told’ that they have an infection and will receive an antimicrobial (often unnamed), leading to loss of ownership, frustration, anxiety and ultimately distancing them from engaging with decision-making. This poor communication drives individuals to seek information from alternative sources, including online, which is associated with concerns over reliability and individualisation. Failures in communication and information provision by clinicians in secondary care influence individuals’ future ideas about infections and their management. This alters their future actions towards antimicrobials and can drive prescription non-adherence and loss to follow-up.

**Conclusions:**

Current infection management and antimicrobial prescribing practices in secondary care fail to engage patients with the decision-making process. Secondary care physicians must not view infection management episodes as discrete events, but as cumulative experiences which have the potential to shape future patient behaviour and understanding of antimicrobial use.

**Strengths and limitations of this study**

- This study adds to the paucity of evidence surrounding the patient experience of infection management in secondary care pathways.
- Our findings provide evidence to support development of interventions to address identified failures of information provision and communication with patients locally.
- This study was an in-depth investigation of a small number of individuals who have been managed for infections within secondary care pathways over the last 12 months.
- Findings were tested with a separate cohort of 20 citizens for validation; this work will facilitate the development of targeted interventions to address the challenges identified within our initial study.

## Introduction

Antimicrobial resistance (AMR) is a global patient health and safety issue, with estimates that up to 10 million deaths each year may be attributable to AMR by the year 2050.^1^ Antimicrobial stewardship (AMS) programmes have been introduced at local and international levels in an attempt to optimise the use of antimicrobials. These interventions aim to achieve the best therapeutic outcomes of treatment, while minimising the harmful consequences of antimicrobial therapy, such as toxicity and development of AMR.^2–8^ To date, most AMS interventions have focused on healthcare providers with current patient engagement interventions around AMR and AMS (mainly via public health interventions) difficult to assess for efficacy.^9–20^

Despite a paucity of evidence to support patient focused interventions within AMS programmes, a growing body of literature is emerging that describes physician's and patient's desire for increased collaboration in the decision-making process surrounding the prescription of medications within secondary care.^21^ However, there is currently no specific evidence describing patients’ experiences of infection management and antimicrobial prescribing within this setting. Within primary care, the role of shared decision-making (SDM), where patients and clinicians come together, acknowledge that there is a decision to be made (ie, between treatments and including no treatment), and consider the best available evidence with the patient's values, preferences and context have been demonstrated to reduce the rates of antimicrobial prescribing for respiratory tract infections.^22^ However in secondary care, where infections are often more serious, requiring urgent and highly protocol driven management, the role for the patient in this process remains unclear.

The aim of this qualitative study was to investigate patients’ current experiences of infection related decision-making processes across secondary care pathways and map how these experiences influence future engagement with infection management and antimicrobial use. Through generalisation of our findings from this in-depth investigation we hope to inform future patient-focused interventions to address the issues identified and allow assessment of their impact on patient outcomes and AMR within secondary care pathways.

## Method

### Participant recruitment

In September 2015, 14 members of the public who had received antimicrobials from a secondary care pathway in the preceding 12 months in the UK were identified for recruitment (through Cherry Picked, London*,* UK; a specialist qualitative recruitment service)*.* This involved identifying a sample of 500 individuals who lived in South-East England and met recruitment criteria (box 1) from a database of 20 000 citizens who had previously signed up to the recruitment service from around the UK. The sample cohort of 500 were contacted with an initial recruitment email to identify those available to take part in the focus group sessions. From there, participants were then stratified according to recruitment criteria and 14 selected based on their fit with the criteria and availability for the session. Two further contacts were made with identified participants following this to confirm their participation and provide directions to the venue. Four individuals declined to participate, giving no reason for this.

Box 1 **Selection criteria for participation in in-depth focus group analysis of current experience of patient engagement with infection management and antimicrobial prescribing across secondary care**

- Age 18 or older.
- Equal gender mix.
- Representative mix of ethnic backgrounds.
- Must have been treated with antibiotics in the secondary care setting (this could include, outpatients, Emergency Departments, Urgent Care Centres or Ambulatory units) within the last 12 months. This should not have been level 2 or 3 care (eg, high dependency units or intensive care) only.*
- Preferable that they have been an inpatient in secondary care previously (but not an exclusion criterion if the above criteria are all satisfied).

*Individuals receiving antimicrobials in level 2 or level 3 care facilities only were excluded, given that they are likely to have been critically ill at the time of antimicrobial prescribing.

Participants attended focus group interviews at Imperial College London (UK). A small sample size was selected in order to gain an in-depth understanding of individuals’ views, thus providing a richness to the data available for analysis.^23^ Furthermore, focus groups were selected over individual interviews as these allowed for group exploration of new ideas, point-counterpoint discussion and resolution of views; allowing identification and consensus on common themes within the groups.^23^ All individuals were consented prior to participation. Participants completed a questionnaire collecting demographic data and previous healthcare experiences. The validated Single Item Literacy Screener (SILS) screening tool was included to assess the participant's level of health literacy,^24^ to allow estimation of the group’s rate of health literacy and comparison to that of the general population. This was felt to be important for consideration, given that the findings of this study may be used to inform future interventions in clinical practice. A reimbursement of £65 (US$100) was provided to participants for their time.

### Participant focus groups

The group was divided into two equal groups based on age categories and gender. Two healthcare professionals (TMR, LSPM), following a predetermined schedule (see online supplementary data 1; developed from a critical analysis of the literature), facilitated a 120 min focus group. This aimed to explore the participants’ experiences of engagement with decision-making surrounding infection management and antimicrobial use in secondary care pathways. Two independent observers (one lay and one healthcare professional; BH and EC-S) directly observed the sessions and were asked to make notes of key observations. These were used to help triangulation of initial codes during analysis.

10.1136/bmjopen-2016-011040.supp1 supplementary data

### Data analysis

Focus groups were audio recorded and transcribed verbatim (using anonymous participant identifiers). Thematic analysis of transcripts was performed using a mixed deductive and inductive approach.^25^ Deductive categories were identified based on review of the literature and findings from previous work exploring the user’s role in infection control.^26^ For the inductive approach, two authors (TMR and LSPM), reviewed the focus group transcripts independently to allow initial codes to be generated from differing viewpoints by line-by-line coding for first order codes.^27^ ^28^ During line-by-line coding, the comments provided by the independent observers’ were considered with the aim of complementing areas of reflexivity caused by the analysts’ own prior experiences.^29^ After familiarisation with the transcripts, the researchers independently coded the data generating a list of emerging categories from the first order codes and those identified deductively, addressing the aims of the study design. After meeting and agreeing on key categories and themes within the text, the two analysts independently proceeded to systematically cross-review the text, coding passages based on these agreed codes and categories, subsequently grouping them into overarching themes. On review, any discrepancies were discussed and consensus reached. Examples of key opinions and ideas from the text for each main theme identified were then charted to allow mapping and interpretation of the results.^27^ Following synthesis of our findings, 20 new participants were recruited using the same recruitment agency (Cherry Picked, UK) in May 2016 to take part in three further focus group sessions. As a part of these sessions the findings from the initial focus groups were tested for validation within a new group of citizens (data not shown). Through this exploratory work it was deemed that saturation of key categories and themes, identified in the original focus group sessions, had been reached; allowing for progression onto the development and impact of specific interventions that addressed our findings to be explored.

### Ethics approval

The study protocol was reviewed by the West London Regional Ethics Committee (REC) and considered to meet criteria for monitoring under service evaluation governance structures *(REC 15/LO/1269/ICHNT Service Evaluation SE113*).

## Results

The median age of participants was 52 (21–69) years with an equal gender divide. Seven of the participants were of white ethnicity. Six participants had experience of infection management as a hospital inpatient (in the non-critical care setting), with the remaining participants all having received antimicrobials from other secondary care pathways across a variety of South-East England healthcare institutes. These included the Emergency Department (ED), urgent care centres (UCC's) or consultant led outpatient clinics. Two out of ten participants were identified on screening as potentially having a low health literacy, reporting that they sometimes, often, or always required help with written health information on the SILS screening tool.^24^ This indicates that our cohort are likely to be more health literate than the average population, where ∼43% of individual citizens would require assistance with written health information.^24^ ^30^

**Figure 1:**
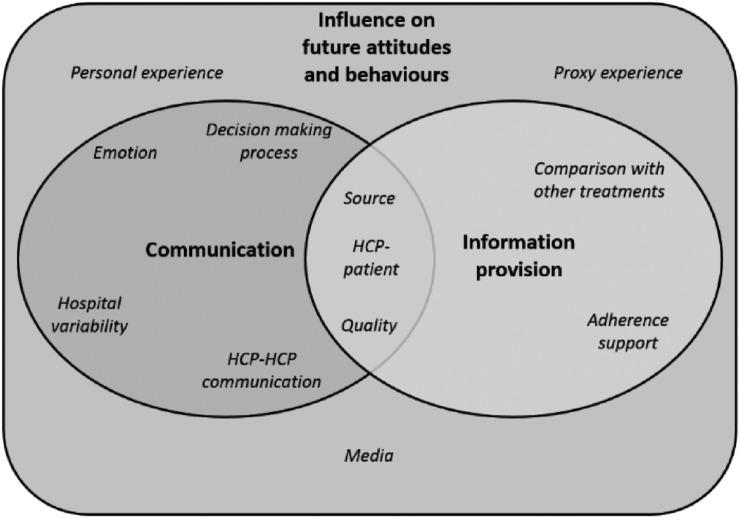
The distribution of themes and categories contributing to individuals’ experiences of decision making for infection management in secondary care pathways.

Following thematic analysis, 92 subcategories that fell into 12 categories were derived from the transcripts. Three interlinking themes were identified (figure 1). Table 1 summarises key quotes informing the individual categories and themes referred to within the text below. The participants described a failure in communication and information provision from clinicians and support staff in secondary care, which subsequently influences the individual's future ideas about infections and their management. This alters the individual's future actions towards infections and antimicrobials and can drive non-adherence to prescribed antimicrobial regimes and loss to follow-up after discharge from secondary care.

**Table 1:** An analytical framework developing categories and themes for patients’ experiences of infection management in secondary care

Quote	Category	Theme
“I wasn't given any education into what to do [with my antibiotics]. The 5^th^day I felt well and so thought I would just stop taking the treatment. I was fortunate that my sister explained to me and made me complete the course” [24-year-old female]	Adherence support	Information provision
“Especially I think that you are often given more information when you are taking other medication… I have allergies to penicillin so always I have to know what kind of antibiotic I have been given. So unless your issues are more complicated, that's when they give you more information, otherwise I feel that they don t provide you with enough” [24-year-old female No. 2]	Comparison with other treatments	
“I like to go and see the doctor… Online can't see me [sic]. Infection is a thousand different things and online can't confidently tell you, this is what you have…” [65-year-old male] “…you are not an individual to them [corporate pharmacists]. In our case, I think we have the option to be sort of individuals. That is what I find lovely about our current pharmacy!” [69-year-old male]	Sources	Information provision/communication
“I think what the problem that I have experienced is, is that they will give you a leaflet to read and I will have to go and research it myself. This is rather than the doctor taking the time to sit down and talk about how it might affect you, what exactly is in it [the antibiotic]—you know a proper consultation.” [23-year-old female] “Rather than sitting down and taking the time to explain, because they use a lot of medical terminology that I do not know what they're talking about to be honest. I think that they need to take more time to be honest to sit down and make sure that the patient knows exactly what they are putting in your body and exactly what all the side effects were. Because I didn't know what I was reacting to…” [24-year-old female]	Quality	
“I think sometimes the doctors normally come and diagnose you they usually tell…. They don't necessarily tell you what they are giving you, they usually prescribe it. Then the nurse just comes along with a pot full of drugs and you just take them. I think, unless you are intrigued and ask for it then the nurse will give you that information.” [30-year-old female]	HCP—Patient communication of information	
“When you go into hospital, you feel as though the illness is not yours. You go in to hospital and everyone takes over, like ‘we do this then we do that later’. You have no ownership in a way. You are going through it but you have no ownership over what is being done for you or what medication you are receiving.” [23-year-old female]	Decision-making process	Communication
“Tell me yes or tell me no… If you can't fix it I don't want to see you again because there will be no point… We've tried this it's not worked so we tried that… it is endless…” [65-year-old male]	Emotion	
“You know, the hospitals I have experienced in [region]—I am not really keen based on the lack of information. It is more about; we're doing this operation—get you in, get you out.” [23-year-old female]	Hospital variability	
“When I went to A&E I visited my GP … It is more about telling your GP what the symptoms were and what treatment you had rather than exactly what the infection is” [30-year-old female] “My GP never knew anything. She had scheduled me in to have the hernia, but the appendix went first. And she was “oh have you…” [53-year-old male]	HCP—HCP communication of information	
“For me, I do not know the difference between an allergy and side effects. I would normally just try and cope with it and not go back to the doctors.” [24-year-old female] “I left it a long time and then I got an infection tracking all the way up [my leg]. I went into A&E as I couldn't walk. When I was there they brought some student doctors and said “how bad is this leg” and I thought [this is bad]!” [60-year-old male]	Personal experience	Influence on future attitudes and behaviours
“They asked whether he was allergic and I said that I do not know he had never had them. After being given them he really severely reacted. He blew up with vomiting and was very very sick. We had to go back to casualty and get that sorted. So the thing that worries me about that is that I remember someone telling me that if you routinely have an operation, you are given penicillin routinely so it worries me whether that would have an effect if he was ill abroad…” [52-year-old female]	Proxy experience	
“I read an article a while ago about antibiotics and how they made people severely ill. A few people have died. I think it's just like… where I have heard about bad experiences…. you know they have never really pulled through for me.” [21-year-old female]	Media	

This data is an extract of quotes derived from thematic analysis of focus group interviews exploring participants’ experiences of infection management in secondary care pathways.

A&E, accident and emergency department; GP, general (primary care) practitioner; HCP, healthcare professional.

### Failures in communication

Participants described their experiences of being diagnosed with an infection in secondary care as one where they completely lost ownership of their condition. Control of their illness was taken over by a multitude of healthcare professionals (HCPs). Recurring instances were identified where HCP communication with patients became unilateral when antimicrobial decisions were being made, with patients being ‘told’ information, often devoid of key aspects such as names of medications, durations of treatment and prospective plans about time courses and potential escalation/de-escalation of therapy. This led to a significant amount of anxiety and frustration as the individual searched for answers.

I was told ‘you have an allergy [to penicillin], take this instead’—Tell me what I am taking and exactly what it is going to do for me! [65-year-old male]

Moreover, in many cases participants did not feel as if they were involved in the decision-making process around their infection management with two-way communication with healthcare professionals perceived as absent.

Apart from HCP communication with patients, participants reported becoming frustrated by communication between HCPs. This is centred primarily on the way in which information about infections is communicated from secondary care doctors to primary care doctors on discharge from the hospital. While patients are provided with a discharge summary of their stay on leaving the hospital, it was perceived that this often neglected information about their infection and the treatment which they received while in the hospital. Participants reported that they were often forced to communicate this information directly with their primary care physician on follow-up visits or were otherwise lost to follow-up after discharge due to lack of clear communication pathways.

### Failures in information provision

The current volume and quality of information provided to individuals by HCPs in secondary care causes problems for patients as it is often poorly explained, with medical terminology routinely used. This leads to a feeling of disempowerment with individuals frustrated that they then have to ‘go away and research it [their condition] themselves’ (23-year-old female). Fear and anxiety follows when participants see serious side effects of treatment ‘like risk of death [and] no one has mentioned that to me!’ (30-year-old male). This in turn causes frustration as participants compare delivery of information on infections and antimicrobials to that provided for operations and medications for chronic disease, such as hypertension. In this example, patients are provided with explanations of their procedure/condition, their management, and potential complications which may arise and how these will be dealt with. In contrast, information on infection management is seen as a ‘reactive’ process where information is only often provided once complications have occurred. Furthermore, patients are often unaware of the timeline for their treatment and the potential complications. This lack of clarity drives individuals to stop treatments early or potentially ignore side effects experienced due to false assumptions and misinformation.

Participants reported that this failure in communication about infections and antimicrobials drives them to seek information from a wide range of sources, often with varying degrees of quality. Participants commonly sought information independently due to ‘difficulties in accessing [healthcare professionals]’ and the ‘[time] pressures of work and children’ (65-year-old male). A number of avenues were preferred such as the Internet, information leaflets provided with medications and local pharmacies. Individuals will seek out recommended or official NHS sources of information which they believe that they can trust to provide them with information on their infection or treatment. While these sources are seen as helpful, patients still prefer to discuss their infection and its management with a HCP as this provides ‘individualised’ information compared to the ‘standard-reply’ provided by alternative sources (69-year-old male). This is because the information provided is seen as being based on the patient's own specific situation and issues. Furthermore, the HCP is a ‘trusted’ source being viewed as an ‘expert’ (69-year-old male).

### Influences of future attitudes and behaviours

Participants clearly described how these individual experiences of poor communication and information provision influence their future ideas and actions towards infection management both in secondary care and in the community. Influences were described from three sources; personal understanding/experiences, understanding by proxy and understanding through the media.

For example, one personal experience was described by a participant who was told that he had an allergy to penicillin and told that he would be given a ‘weaker’ type of antibiotic for his infection. When this was perceived not to be effective at clearing up the infection after 2 days, he stopped taking his medication as:

You know the weaker ones [antibiotics] never seem to clear the infection up. They are not as strong so they don't clear it up. The infection lasts longer [60-year-old male]

This subsequently led to the participant having to return to secondary care for further treatment of his infection due to the poor information provision and engagement in the decision process surrounding his infection.

The media's role in developing the participants’ understanding of infection management arose and was further explored during the focus group. Participants reported that the media’s influence occurred through the portrayal of stories about complications of treatment and the dangers of AMR. This created fear and mistrust of medical professionals within our participant group, and caused participants to be ‘cautious’ when interacting with medical professionals as they are perceived to ‘not say the full story’ (21-year-old female**).** This distrust was reported as driving non-adherence to therapy in the community by several members of the group.

## Discussion

### Summary of participant impressions

Within our participant group, individuals felt detached, frustrated and disempowered from involvement in decision-making about their own infection management within secondary care. The consequences of the failure of HCP communication and information provision reached beyond secondary care, influencing the ideas and actions towards infections and antimicrobials during future healthcare interactions along a number of different pathways. This fosters feelings of frustration and anxiety during an individual's journey through complex secondary care pathways and potentially drives non-adherence to prescribed antimicrobial regimes and loss to follow-up after discharge. These findings highlight the need for specialists in secondary care to not view infection management episodes as discrete events, but as cumulative experiences which have the potential to drive future non-adherence to prescribed antimicrobial regimes and thus the promotion of AMR.

### Opportunities for educating healthcare providers to improve patient engagement

Importantly, HCPs must appreciate that engagement in the decision process for infection management and antimicrobial prescribing may have an influence on future patient actions towards infections and antimicrobial use. These actions can be influenced by personal experience along with those of friends and family and what is described in the media. The way in which we communicate information to patients was reported as the most important aspect in our participants’ experience of infection management in secondary care and was the largest influence on future actions in terms of adherence to prescribed antimicrobial regimes and healthcare seeking behaviours. Participant perception of communication in secondary care infection-related pathways is of a unilateral process which does not invite patient participation. Greater emphasis needs to be placed on educating HCPs to move away from the decision-maker role^31^ into a more bilateral structure. Difficulties such as time pressure on the HCP and the patient is perceived as a key factor by participants and must be taken into account when designing interventions to help facilitate improved communication and patient education during the decision-making process. The way that these interventions are designed must be mindful of health literacy, ensuring that the information provided to patients is understandable. Within our small cohort, two of the ten participants met the screening criteria for health illiteracy. Within the UK, it is estimated that up to 43% of the adults cannot understand currently available health information.^24^ ^30^ Therefore, along with educating healthcare providers in how to improve communication with patients, consideration of the wording and type of health information supporting this is vital to allow patient engagement with the decision-making process.

### Opportunities for improving patient engagement with decision-making

Within our cohort, participants felt strongly that the choice of information provided about their infection and antimicrobial therapy should be dictated by the patient's preference. However, their focus was not primarily on the end decision of whether or not to treat, but on feeling involved and engaged with the process of decision-making. This focused on education about their condition and treatment, communicated effectively to them. They described a belief that if a trusted clinician felt they had an infection that required antimicrobial therapy, then this was appropriate. Whether this is truly sharing the decision process or not is for consideration, as SDM classically acknowledges that there is a choice to be made, with the patient and clinician coming together to consider available evidence, the patient’s values and preferences before arriving at a decision.^32^ However, Edwards and colleagues suggest that this can still be classed as sharing the decision (or engaging the patient in the process) where the focus is placed primarily on involving the patient in the decision-making process, rather than on who actually makes the final decision on management.^33^ Our participants supported this approach to engagement by describing how they become frustrated and distrusting of the recommended therapy when supporting information about the infection and the proposed management is perceived to be withheld from them.

Participants currently view information provided about infections and antimicrobials as reactive in nature, with information only provided after a side effect occurs or the patient fails to respond to a certain type of antimicrobial, and therapy is escalated. Individuals want proactive information to help them understand what they are receiving, what to expect and what the plan is if the treatment does not go to plan. This allows them to feel ‘prepared’, ‘confident’ and invested in the healthcare they are receiving. This is challenging for antimicrobial prescribing in secondary care, which is often an acute event, requiring rapid decision-making, and has a short duration of therapy.^34^ Moreover, this highlights a key area of misunderstanding surrounding infections and antimicrobial therapy within our participant group that has been driven by poor communication and information provision during previous experiences of infection management within secondary care. Therefore, future tools must aim to promote patient engagement with infection management, considering how they define engaging patients in the decision process. Moreover, these interventions must ensure that identified deficiencies in how HCP communicate and provide information to patients are addressed to facilitate improvements in the current patient experiences.

### Strengths and limitations

This qualitative analysis aimed to map the current experiences of patients in antimicrobial decision-making but it does have limitations. Group facilitation within our study was carried out by two HCPs, which may have influenced socially desirable participant responses to certain questions. To address this dynamic between interviewer and interviewee, two observers’ comments were also considered during initial coding to highlight where the interviewer's position may have directly influenced individual responses. For example, during discussion of participants’ perceptions of doctors attitude towards prescribing antimicrobials, one participant apologised after voicing an opinion about doctors simply wanting to

..sign the prescription and get rid of the patient [69-year-old male].

The noted anxiety about offending the HCP may have influenced other participants voicing their true opinion on the matter. Second, while small, this in-depth study provides key themes for future studies to explore the generalisability of and inform the design and evaluation of appropriate interventions. Furthermore, our findings were subsequently tested for validation within an independent group of citizens to search for further categories and themes within our local population. Finally, on comparison of the health literacy of our selected cohort of participants, the group appeared to be more health literate than estimates for the general population. Therefore, during subsequent intervention development and exploration, this aspect must be highlighted and considered as this may affect the generalisability of our results across the population.

## Conclusion

Within secondary care, specialists are failing to engage their patients with the decision-making process surrounding infections and their management. This ultimately leads to misinformation, frustration and anxiety during an individual's journey through secondary care pathways and potentially drives non-adherence to prescribed antimicrobial regimes and loss to follow-up in the community. Clinicians must stop seeing infection episodes as discrete events and approach them with the understanding that previous negative experiences drive subsequent non-adherence to prescribed antimicrobial regimes and potentially disrupt follow-up of patients post discharge from secondary care. Poor communication by HCPs and lack of quality information provided are the two leading causes for this, often driving individuals to seek standard information from untrusted online sources. This aspect must be addressed through improving HCP education on patient engagement and through development of interventions to support patient engagement in the process. Furthermore, these findings have the potential to translate into other fields of secondary care, where poor engagement also exists and benefits in patient outcomes through interventions promoting improved communication and information provision are beginning to be reported. We call for the development of clear and pragmatic mechanisms to educate HCPs and provide patients with the proactive information they require about their infection and its management and engage them with the decision-making process.
