# Supplementary material for: Patient engagement with infection management in secondary care: a qualitative investigation of current experiences
Source: BMJ Open. 2016 Oct 31;6(10):e011040. doi: 10.1136/bmjopen-2016-011040 (PMC5093376; doi:10.1136/bmjopen-2016-011040)
Supplement: supplementary data [file bmjopen-2016-011040supp.pdf]

Supplementary data 1 - Workshop Topic Guide

- Conducting a service evaluation to understand and improve the process of shared decision making during antimicrobial prescribing by clinicians in secondary care (inc. UCC / OP etc)
- We are interested in your own opinions and perceptions of this problem and not what you think others would want you to say
- Everything is kept confidential and no one within the Trust will know what has been said by you. To ensure that confidentiality is maintained you will be assigned a participant number
- We ask you however, NOT to reveal any specific personal information
- Time limit approx. **(as above)** to complete

Topic	Aims	My Questions
**1. Introduction**	Consent<br><br>Collect baseline demographic data<br><br>Collect individual opinions for triangulation against collective group views	• **Welcome, brief outline of aims of day**<br>• **Why you have been invited**<br>Broad range of people who have been prescribed antibiotics in secondary care (or around) setting<br>Want to explore what information you receive and how you engage with the decision making process<br>• **Consent and baseline questionnaires (confidential).**<br>• **Split into two groups to begin (delegated before session from participant charter provided by company)**
**2. Exploration of current issues during consultations**	Reflect on current level of information provided to patients by clinicians when prescribing antimicrobials<br><br>Reflect on how this information is delivered in different settings (on the wards, admission vs. discharge)<br><br>Explore whether this information is adequate<br><br>Explore whether the participant feels as if they are involved in the decision making process in these scenarios<br><br>Explore barriers to "successful" use of antimicrobials (i) in hospital and (ii) on d/c	• **Can you describe what kinds of information you were provided about the antibiotics you were prescribed last time you were in hospital (or similar) (i) at the point of prescription (ii) at the point of discharge**<br><br>• **How did you receive this information?**<br>**<u>Prompts for above:</u>**<br>*prescription? antimicrobial box insert? Printed information from the GP*<br>*Did you read it?*<br>*Did it give you the information you were looking for?*<br><br>• **Who gave you most of this information?**<br>Prompt: Dr / Nurse / Pharmacist?<br><br>• **Was there anything missing that you would like to have been told / had discussed with you?**<br><br>• **What are the common questions about your infection/antibiotics do you ask your doctor?**<br>Prompt: When do you ask these (during or after reflection)?<br><br>• **Did you feel as though you were a part of the decision making process when you were you and your doctor discussed your infection / treatment?**<br>1. Can you explain why you felt this?

<table>
<tr><td></td><td>with antimicrobials</td><td>What extra information do you seek independently following discussion with the doctor?
Prompt: Is this because:
There is not enough time to have questions addressed
The information provided is not clear
The patient has a personal view on allopathic meds?
Your were embarrassed to ask the question?
What are the day to day challenges (i) in hospital (ii) following discharge with adherence to a course of antibiotics
Prompt:
Remembering to take the course / timings / monitoring for s/e's
Do you complete the course?
What do they think is the major barriers to the above?
Prompt:
? Lack of information
? Lack of understanding over importance
? Other
When you visit the GP after a visit to hospital do they know all of the details about the infection & antibiotics that you received during your visit?
How do they receive this information?
Would you be able to explain to the GP which meds you are on and why?
Prompt:
Do you tell them the majority of this info?
If so how do you record it?
Clinic letter / discharge summary? (do you feel they get the full picture from it?)</td></tr>
<tr><td>3. Feedback to group</td><td>Allow group to understand all issues identified during each groups session</td><td>Leads briefly summarise each groups key findings
Allow discussion and consensus on any major points of difference which arise between groups</td></tr>
<tr><td>3. Generating approaches to solving these issues</td><td>Explore what further information patients would like to receive

Explore what other support with antibiotic use patients feel they require</td><td>How do you go about finding information about the infection or antibiotics you are given? (a) during your hospital stay (b) once your are discharged with them?
On attaining this information do you feel that it helps you participate more actively in discussions about your infection / antibiotic treatment with the doctors and other HCP's?
Prompt:</td></tr>
</table>

	Explore how patients currently acquire this information which they perceive as helpful / whether they receive this support<br><br>Does this information empower them to take an active role in their infection / antimicrobial therapy<br><br>Explore approaches that patients would like to be available to attain this information<br><br>Investigate whether any other support would be helpful	Do you feel as if you are involved in the decision making?<br>Do you feel that your views and ideas are considered?<br><br>• **Is there any difference in the information you require on this (i) when in the hospital c.f. (ii) at the point of discharge on antibiotics**<br>Prompts:<br>Do you look up things the doctor tells you about your infection / antibiotics?<br>Is this more helpful in or out of hospital?<br><br>• **Are there any other measures that would be helpful in helping you better understand your infection / support you in taking the course of antibiotics?**<br><br>• **Brain storm ideas of how patients could receive / access this information / support - Rank using nominal group tech.**<br><br>• **Consensus through discussion**<br>Prompt:<br>(a) Route of info (app / email / text message / interactive / recording / webcast / paper based)<br>(b) What would be provided<br>(c) Level of detail<br>(d) Original info? Reputable source ? individuals own experiences (uncensored)<br>(e) Ideal timing to receive this information
**4. Triangulate**	Confirm results generated through each group<br><br>Explore whether there are any other comments / observations participants wish to make	• **Present both groups nominal group exercises**<br>• **Summarise similarities and differences**<br>• **Does anyone wish to discuss these?**
